# Effect of preconceptional orlistat treatment on in-vitro fertilization outcome in overweight/obese women: study protocol for a randomized controlled trial

**DOI:** 10.1186/s13063-018-2780-7

**Published:** 2018-07-18

**Authors:** Jing Li, Ze Wang, Daimin Wei, Hong Liu, Jiangtao Zhang, Jianfeng Wang, Yuhua Shi, Zi-Jiang Chen

**Affiliations:** 1 Center for Reproductive Medicine, Shandong Provincial Hospital Affiliated to Shandong University, Key Laboratory of Reproductive Endocrinology, Shandong University, Ministry of Education, and National Research Center for Assisted Reproductive Technology and Reproductive Genetics, Jinan, China; 2 Center for Reproductive Medicine, Ren Ji Hospital, School of Medicine, Shanghai Jiao Tong University, Shanghai Key Laboratory for Assisted Reproduction and Reproductive Genetics, Shanghai, China

**Keywords:** Orlistat, Overweight, Obese, Randomized controlled trial, Live birth

## Abstract

**Background:**

Obese women have fewer oocytes retrieved, an increased cancelation rate, a higher miscarriage rate, and a lower live birth rate after assisted reproductive technology (ART) treatment compared with women with normal weight. Weight loss before ART treatment can significantly improve pregnancy rates and/or live births. An orlistat plus diet intervention could promote weight loss, but there is no evidence from randomized clinical trials evaluating the effect of orlistat preconceptional treatment on pregnancy outcome in overweight and obese women.

**Methods/design:**

We are conducting a multicenter, randomized placebo-controlled, double-blind clinical trial in overweight and obese women aged 20–40 years undergoing in-vitro fertilization and embryo transfer (IVF-ET) with or without intracytoplasmic sperm injection, to evaluate whether orlistat treatment for 1–3 months before IVF-ET can improve the live birth rate. The primary outcome is live birth.

**Discussion:**

The results of this study will provide evidence for the effect of preconceptional orlistat treatment on IVF outcome in overweight/obese women.

**Trial registration:**

Chinese Clinical Trial Registry, ChiCTR-IPR-17011629. Registered on 11 June 2017.

**Electronic supplementary material:**

The online version of this article (10.1186/s13063-018-2780-7) contains supplementary material, which is available to authorized users.

## Background

The prevalence of obesity is increasing worldwide, and it causes not only an increased risk of metabolic syndrome, type 2 diabetes, and cardiovascular disease, but also a decline in fertility and an increased risk of infertility [1]. Obese women have a higher dose of gonadotrophin, more days of stimulation, fewer oocytes [2, 3], an increased cancelation rate, a higher miscarriage rate [4], and a lower live birth rate [5, 6] compared with women with normal weight. According to a systematic review by Maheshwari et al. [7], women with BMI ≥ 25 kg/m^2^ have a lower chance of pregnancy and an increased miscarriage rate following IVF. Another meta-analysis showed similar results that women with BMI ≥ 25 kg/m^2^ had significantly lower live birth rates compared to those with BMI < 25 kg/m^2^ following IVF/ICSI treatment [8]. The risk for preeclampsia and gestational diabetes was doubled in obese pregnant women [9], and maternal obesity was associated with an increased risk of many structural anomalies [10, 11]. Short-term weight loss was associated with higher metaphase II (MII) oocyte yield, but unrelated to clinical pregnancy or live birth rates [12]. Another study reported that a “meaningful” weight loss of 10% could significantly improve live birth rates (71% vs 37%) [13]. A systematic review showed that women with weight loss before ART treatment had significantly improved pregnancy rates and/or live births [14].

A high-caloric diet is the main cause of obesity, and a higher fat intake is associated with dyslipidemia and insulin resistance. The common strategy to lose weight includes modification of unhealthy dietary habits, more physical activity, pharmacological management, and bariatric surgery. But poor patient compliance with lifestyle modification is known because it needs a long time to take effect. Therefore, it is reasonable to focus on the consumption of fat to lose weight.

Orlistat prevents the absorption of triglyceride from the human diet by irreversibly combining with gastric and pancreatic lipase, and it is reported that an orlistat plus diet intervention could decrease weight, cholesterol, and insulin levels [15, 16]. But there is no report about the effect of orlistat before ART treatment on pregnancy outcome in overweight and obese women.

## Methods/design

### Design and setting

This study is a multicenter, randomized placebo-controlled, double-blind clinical trial to evaluate whether orlistat treatment for 1–3 months before IVF-ET can improve the live birth rate. Patients are recruited from 19 hospitals in China. In this study, overweight is defined as BMI ≥ 25 kg/m^2^ and obesity as BMI ≥ 28 kg/m^2^ according to Chinese criteria.

### Inclusion criteria

The inclusion criteria are as follows:

1. Women aged ≥ 20 and ≤ 40 years.
2. Women with BMI ≥ 25 kg/m^2^.
3. Women who are undergoing IVF or intracytoplasmic sperm injection (ICSI).

### Exclusion criteria

The exclusion criteria are as follows:

1. Women who had no clinical pregnancy after three or more cycles of IVF/ICSI.
2. Women who have been diagnosed with congenital or acquired uterine abnormalities by ultrasound or with severe intrauterine adhesion by hysteroscope.
3. Women with abnormal liver or renal function.
4. Women with an allergy to orlistat.
5. Women with a diagnosis of malabsorption syndrome or cholestasis.
6. Women with organic obesity; for example, hypothyroidism.

### Sample size

Based on the retrospective data from our IVF clinic, the live birth rate of obese or overweight women is about 35%. It is assumed that an improvement of 10% in the live birth rate will be clinically significant. We aim to test a difference of 10% in the live birth rate between treatment groups (45% in the orlistat group) at a significance level of 0.05 with a statistical power of 80%. The minimal sample size calculated is 752, and 836 participants will be enrolled in consideration of a dropout rate of 10%.

### Screening

**Fig. 1:**
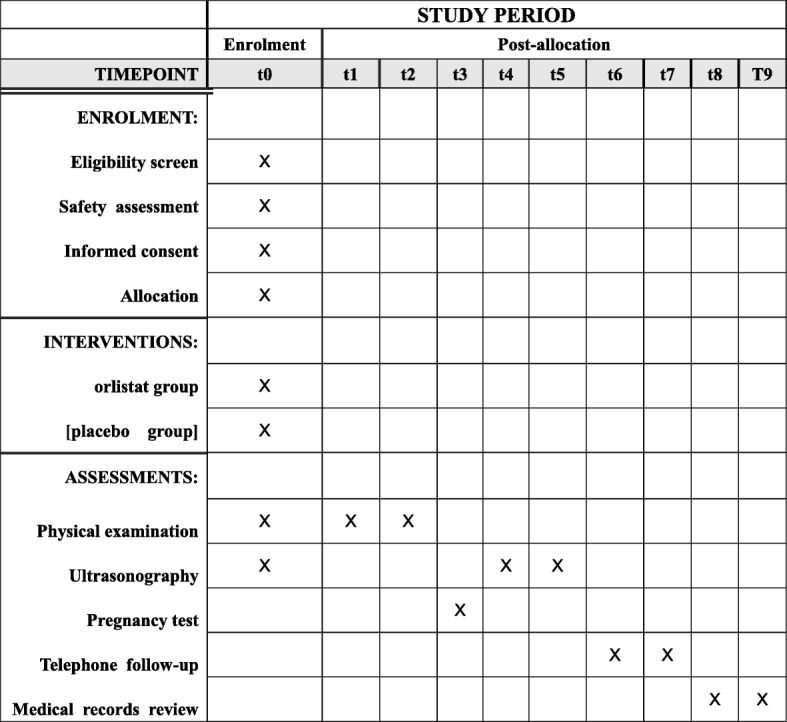
SPIRIT diagram for schedule of enrollment, interventions, and assessments. Safety assessment includes fasting blood glucose, insulin, TG, TC, HDL, LDL, Lp (a), liver function, renal function, CBC, coagulation test, HBV, HCV, HIV, syphilis, and routine urine. t1, 1–3 months after allocation; t2, day of ET; t3, 2 weeks after ET; t4, 5 weeks after ET; t5, 10 weeks after ET; t6, 28 weeks of gestation; t7, 37 weeks of gestation; t8, delivery; t9, 6 weeks after delivery

**Fig. 2:**
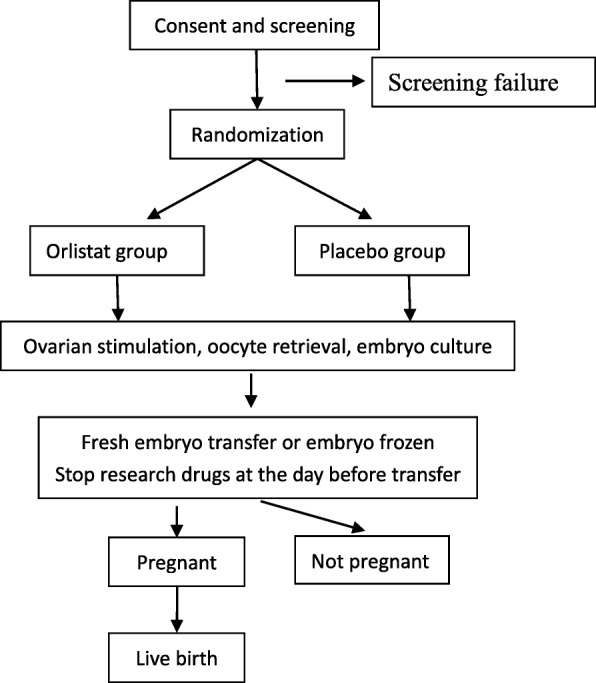
Flow chart of subjects

At the screening visit, patients who have been using orlistat or other weight-reducing aid/drugs will be excluded, and the couples sign the written informed consent after safety assessment, which includes fasting blood glucose, insulin, triglyceride (TG), total cholesterol (TC), high density lipoprotein (HDL), low density lipoprotein (LDL), lipoprotein a (Lp (a)), liver function, renal function, complete blood count (CBC), coagulation test, hepatitis B virus (HBV), hepatitis C virus (HCV), human immunodeficiency virus (HIV), syphilis, and routine urine. The standardized case report forms are completed to collect current medication status and previous medical history. A physical examination (height, body weight, waistline, hipline, blood pressure) and transvaginal ultrasound scan are performed. The basal sex hormones are tested in the local laboratories of the study sites. A diagnostic hysteroscopy is performed in patients who are suspected to have an abnormal uterine cavity after ultrasound scan. All participants are prescribed multivitamins and advised to have a healthy lifestyle, more physical activity, and a reduced-calorie, low-fat diet, and to avoid gaining weight rapidly during pregnancy. A schedule of enrollment, interventions, and assessment is provided in the Standard Protocol Items: Recommendations for Interventional Trials (SPIRIT) figure (Fig. 1). The flow chart of this study is shown in Fig. 2. The SPIRIT checklist is presented in Additional file 1.

### Randomization and blinding

A total of 836 patients will be enrolled, and a computer-generated random list generated by SPSS 16.0 (SPSS Inc., Chicago, IL, USA) will be used for randomization. The packaging is marked according to the random number list; the packaging and tablets of orlistat (Huadong Pharmaceutical Co., Ltd) and the placebo have the same appearance, which cannot be distinguished. The placebo is manufactured by Huadong Pharmaceutical Co., Ltd, and, in addition to the active ingredients, the rest of the excipient and the appearance and odor are exactly the same as orlistat. The random number list is kept strictly confidential by the Data Coordination Committee (DCC) staff, and the researchers who are in charge of the enrollment have no access to the list. Study personnel are blinded to upcoming treatment group allocation. Participants will receive the medication (orlistat or placebo with a 1:1 ratio). The appearance of orlistat and placebo is identical in packaging, and the participants and all research staff do not know the allocation until the end of the study. The quality of the placebo, such as contents and bacteria contaminations, was controlled rigorously according to the GMP standard.

### Interventions

Participants are randomized to receive either orlistat 120 mg p.o. three times daily (intervention) or placebo 120 mg p.o. three times daily after signing consent at the screening visit. Both groups of participants will take orlistat or placebo for 1–3 months before ovarian stimulation and to the day of fresh embryo transfer.

The protocol of ovarian stimulation is decided by the physicians according to the patients’ characteristics. Oocyte retrieval is performed 34–36 h after human chorionic gonadotrophin (hCG) injection by experienced physicians, and oocytes are inseminated by IVF or ICSI according to the quality of sperm. On day 3 or 5 of the embryo culture, the quality of embryos is assessed by experienced embryologists, and the morphological criteria are selected by the local laboratory. No more than three embryos on day 3 or 5 will be transferred, and the rest of the embryos will be frozen according to local experience. The protocol of luteal phase support and the day of starting will be determined by the local center. The serum hCG test is performed after 2 weeks of transfer, and if the test is positive, follow-up will be continued to the end of pregnancy or delivery.

### Outcome and outcome assessments

The primary outcome is live birth after first fresh embryo transfer. The secondary outcomes include changes of body weight, moderate and severe ovarian hyperstimulation syndrome (OHSS), dose of gonadotrophin during ovarian stimulation, numbers of oocytes, embryos of high quality, conception, clinical pregnancy, ongoing pregnancy, miscarriage, pregnancy and perinatal complication, birth weight, neonatal complication, and other adverse events.

Conception is defined with the result of serum β-hCG ≥10 mIU/ml measured 2 weeks after embryo transfer.

Clinical pregnancy is defined as detection of a gestational sac in the uterine cavity by a transvaginal ultrasound scan 20 days after conception confirmation.

An ectopic pregnancy is defined as a complication of pregnancy in which the embryo implants at any site other than the endometrial lining of the uterine cavity.

Ongoing pregnancy is defined as detection of a viable fetus with fetal heartbeat at 11–12 weeks of gestation.

Miscarriage is defined as pregnancies that eventuate in a spontaneous abortion before 20 weeks of gestation.

A live birth is defined as the delivery of any number of newborns at ≥ 28 weeks of gestation with heartbeat and breath.

### Safety assessment

Adverse events (AEs) are any untoward medical occurrences associated with the subject’s participation in the research, which do not necessarily have a causal relationship with the study intervention. Serious adverse events (SAEs) are events that occur during the subject’s participation in research that meet any of the following criteria: death, life-threatening, severe or persistent disability, requiring inpatient hospitalization or prolongation of existing hospitalization, neonatal death up to 6 weeks after delivery, congenital anomaly or birth defect, or any events deemed serious by the local principal investigator.

Participants will be questioned to report all AEs and unintended concomitants medications at each visit, and all reported AEs will be assessed and recorded. All abnormal changes of blood tests will be evaluated by the investigators. SAEs will be reported in 5 days; SAEs which are unintended and possibly associated with interventions will be reported within 24 h.

### Data collection and management

To ensure the accuracy of outcome assessments and data collection, all of the physicians, nurses, and research assistants will attend a training workshop before the start of the trial. All attenders will be provided with a protocol and standard operation procedures, and will discuss the topics they may feel confused about until everyone is totally clear about the procedures. The DCC is responsible for the monitoring tasks of the trial. Personnel of Shandong University will regularly check the data from different sites, determine the issues that affect the quality and speed of research, and take corresponding measures.

After screening and signing of informed consent, the current medication status, previous medical history, height, body weight, waistline, hipline, blood pressure, transvaginal ultrasound scan, and basal sex hormones will be recorded.

OHSS, dose of gonadotrophin, and information on oocytes and embryos will be assessed from the start of ovarian stimulation to the day of the pregnancy test.

Serum β-hCG will be measured 2 weeks after embryo transfer. If the serum β-hCG test is positive, a transvaginal ultrasound scan will be performed 20 days later to detect the gestational sac.

During pregnancy beyond 12 weeks, participants are contacted by telephone call at 28 and 32 weeks to inquire about pregnancy complications, AEs, and combination therapy.

At delivery, obstetrical and perinatal complications and neonatal information are obtained with reference to obstetric medical records and neonatal medical records.

At 6 weeks after delivery, postpartum complications and neonatal complications are followed up by telephone call.

Data are collected and recorded on a standard case report form, and when the visit is completed in every site, all of the recorded data will be entered into the web-based data system by the double-entry method.

Any participant may quit the trial at any time for any reason. If any patients want to quit, clinicians will ask whether they agree to finish the follow-up according to the trial schedule. All patients who quit and are lost to follow-up will be recorded.

### Data analysis plan

The data will be analyzed by SPSS 16.0 (SPSS Inc., Chicago, IL, USA). The analysis will be conducted using intention-to-treat principles. The primary outcome, the live birth rate, will be compared between two groups using the Pearson chi-square test; secondary outcome parameters, such as the pregnancy rate, OHSS rate, and other rates, will be analyzed using the Pearson chi-square test. A per-protocol analysis will be performed according to the actual participants completing the entire trial. Continuous data are expressed as mean ± standard deviation, with Student’s test for data with normal distribution and Wilcoxon rank-sum test for nonnormal distribution. Categorical data are represented as frequency and percentage with chi-square analysis or Fisher’s exact test for expected frequencies less than 5. *P* < 0.05 will be considered significant.

The reasons for missing data will be recorded, and according to these reasons, four approaches may be considered: complete-case analysis, single imputation methods, estimating-equation methods, and methods based on a statistical model.

## Discussion

This is a trial evaluating the effect of preconceptional orlistat treatment on pregnancy outcome in overweight and obese women aged 20–40 years after IVF. We plan to enroll 836 subjects from 19 hospitals in China. The enrollment began in July 2017. At the time of manuscript preparation, more than 350 subjects have been enrolled. The result of this multicenter randomized trial will provide valid evidence for the effect of orlistat pretreatment on IVF pregnancy outcome in overweight and obese women. We speculate that taking orlistat before ART treatment may improve the pregnancy outcome in overweight and obese women.

### Trial status

The enrollment is ongoing at the time of manuscript submission.

## Additional file

Additional file 1: SPIRIT checklist. (DOC 125 kb)

## Abbreviations

AE: Adverse event
ART: Assisted reproductive technology
BMI: Body mass index
CBC: Complete blood count
DCC: Data Coordination Committee
hCG: Human chorionic gonadotrophin
HDL: High density lipoprotein
ICSI: Intracytoplasmic sperm injection
IVF: In-vitro fertilization
LDL: Low density lipoprotein
Lp (a): Lipoprotein a
OHSS: Ovarian hyperstimulation syndrome
SAE: Serious adverse event
TC: Total cholesterol
TG: Triglyceride
